# Characterizing coinfection in children with COVID-19: A dual center retrospective analysis

**DOI:** 10.1017/ice.2020.1221

**Published:** 2020-09-23

**Authors:** David D. Zhang, Mary Ellen Acree, Jessica P. Ridgway, Nirav Shah, Aniruddha Hazra, Urmila Ravichandran, Madan Kumar

**Affiliations:** 1 Department of Pediatrics, Section of Pediatric Infectious Disease, University of Chicago Medicine, Chicago, Illinois; 2 Division of Infectious Disease, NorthShore University HealthSystem, Evanston, Illinois; 3 Department of Medicine, Section of Infectious Disease and Global Health, University of Chicago Medicine, Chicago, IL

*To the Editor*—Understanding the prevalence of coinfections with coronavirus disease 2019 (COVID-19) is crucial to delineating its true clinical impact. Numerous studies have evaluated coinfections in adults with COVID–19,^1-3^ but data on pediatric COVID-19 coinfections are limited. Here, we evaluate the burden of coinfections in pediatric COVID-19 patients at 2 large Chicagoland medical centers.

## Methods

We retrospectively reviewed electronic health records of all pediatric patients tested for severe acute respiratory coronavirus virus 2 (SARS-CoV-2) from March 9, 2020, through April 30, 2020, in 2 Chicagoland medical centers. At the University of Chicago Medicine, SARS-CoV-2 was diagnosed using one of the following real-time reverse transcriptase polymerase chain reaction (RT-PCR) assays on respiratory specimens: Cobas SARS-CoV-2 RT-PCR assay (Roche Basel, Switzerland) or Xpert Xpress SARS-CoV-2 test (Cepheid, Sunnyvale, CA). Respiratory coinfections were primarily identified using a multiplex RT-PCR respiratory viral panel (RVP) with the following targets: adenovirus, coronavirus 229E/HKU1/NL63/OC43, human metapneumovirus, influenza-A/-B, parainfluenzas 1–4, respiratory syncytial virus, *Mycoplasma pneumoniae*, *Chlamydophila pneumoniae*, *Bordetella pertussis*, and rhinovirus/enterovirus (FilmArray Respiratory Panel, BioFire Diagnostics, Salt Lake City, UT). Coinfections were also identified using the influenza/respiratory syncytial virus (RSV) RT-PCR assay (Cepheid Xpert Xpress Flu/RSV) known as the influenza/RSV panel (IRP). At NorthShore University HealthSystem, SARS-CoV-2 was identified similarly using RT-PCR: Xpert Xpress or BD Max (Becton Dickinson, Franklin Lakes, NJ). Coinfections were detected using a multiplex RT-PCR panel that contained only the viral targets of the RVP (GenMark Dx, GenMark Diagnostics, Carlsbad, CA), as well as an IRP (Roche Cobas Liat Influenza A/B and RSV). We included all RVPs and IRPs that were obtained within 7 days of a SARS-CoV-2 test.

We also reviewed antibiotic prescriptions within 7 days of a positive SARS-CoV-2 RT-PCR result, and we evaluated antibiotic indication to confirm whether bacterial coinfection was present. Combined means, frequencies, and standard deviations were calculated from the 2 subgroups of data. The Fisher exact test was used to detect any significant differences in proportions of coinfection between the SARS-CoV-2 positive and negative groups. Statistical significance was defined as *P* < .05. Stata version 16 software (StataCorp, College Station, TX) was used for all analyses.

## Results

During the study period, 3,669 specimens were sent for SARS-CoV-2 testing, and 862 of these (23.4%) were positive. Furthermore, 767 (20.9%) specimens had a paired RVP or IRP within 7 days. Of these paired specimens, 101 (13.2%) were positive for SARS-CoV-2. The average ages of our aggregate pediatric COVID cohort and the coinfected subgroup were 17.1 years (standard deviation 5.79) and 17 years (standard deviation, 5.11), respectively.

Analyses between RSV/influenza and the rest of the respiratory viral pathogen panel were conducted separately given the large number of IRPs performed (ie, 351 total multiplex RVPs and 424 total RSV/influenza IRPs). Only 2 paired specimens containing the RVP (12.5%) tested positive for SARS-CoV-2 and an additional respiratory pathogen (Table 1). In 1 of these 2 patients, 2 viral pathogens on the RVP (rhino/enterovirus and adenovirus) were detected. Of those who tested negative for SARS-CoV-2 who had the RVP, 130 (38.8%) tested positive for at least 1 pathogen, excluding RSV and influenza. The most common pathogen isolated was rhino/enterovirus (23.3%). Specimens positive for SARS-CoV-2 with a paired RVP were significantly less likely to be positive for any other respiratory pathogen than specimens that were negative for SARS-CoV-2 (*P* = .036). Only 2 (2.0%) paired specimens tested positive for both SARS-CoV-2 and either RSV or influenza, whereas 39 paired specimens (5.9%) that were positive for either RSV or influenza tested negative for SARS-CoV-2 (Table 1). No association was found between individual respiratory pathogens and SARS-CoV-2 status.

**Table 1.** Proportions of Positive Respiratory Viral Pathogens Stratified by SARS-CoV-2

Variable	SARS-CoV-2 Positive, no. (%)	SARS-CoV-2 Negative, no. (%)	*P* Value^b^
**Patients undergoing multiplex RVP testing^a^**	(n = 16)	(n = 335)	
Positive for any respiratory pathogen	2 (12.5)	130 (38.8)	.036
Adenovirus	1 (6.3)	24 (7.2)	
Coronavirus 229E	0 (0.0)	2 (0.6)	
Coronavirus HKU1	0 (0.0)	2 (0.6)	
Coronavirus NL63	0 (0.0)	11 (3.3)	
Coronavirus OC43	0 (0.0)	5 (1.5)	
Human metapneumovirus	0 (0.0)	25 (7.5)	
Parainfluenza 1	0 (0.0)	2 (0.6)	
Parainfluenza 2	0 (0.0)	2 (0.6)	
Parainfluenza 3	0 (0.0)	1 (0.3)	
Parainfluenza 4	0 (0.0)	3 (0.9)	
*Mycoplasma pneumoniae*	0 (0.0)	0 (0.0)	
*Chlamydophila pneumoniae*	0 (0.0)	0 (0.0)	
*Bordetella pertussis*	0 (0.0)	1 (0.3)^c^	
Rhino/enterovirus	2 (12.5)	78 (23.3)	
**Patients tested for RSV and influenza**	(n = 101)	(n = 666)	
Positive for RSV or influenza	2 (2.0)	39 (5.9)	.151
Influenza A	1 (1.0)	4 (0.6)	
Influenza B	0 (0.0)	12 (1.8)	
Respiratory syncytial virus	1 (1.0)	25 (3.8)	

Note. RSV, respiratory syncytial virus; RVP, respiratory viral panel.

a RSV and influenza analyzed separately given large volume of RSV/influenza (IRP) tests.

b Statistical significance determined by the Fisher exact test. Every individually stratified respiratory pathogen had *P* > .05.

c Bacterial targets were not included in a minority of multiplex RT-PCR tests performed on GenMark Dx. The denominator for these targets is 323.

Among all pediatric patients who tested positive for SARS-CoV-2, without respect to having a paired RVP/IRP, there were 35 patients (4%) who received antibiotics within 7 days before and after the SARS-CoV-2 assay. Among these patients, only 8 of 35 COVID-19 patients (23%) receiving antibiotics had an indication of lower respiratory tract infection (LRTI), and streptococcal pharyngitis (12 of 35, 34%) was the most common non-LRTI indication.

## Discussion

Recent reports have shown pediatric COVID-19 coinfection rates as high as 51%.^4,5^ However, our dual-center study revealed that viral coinfection rates in pediatric COVID-19 patients are low. This analysis was performed at a time of year when respiratory viral transmission, most notably influenza, was declining. During the study period, the Illinois Department of Public Health tracked a decrease in positive influenza tests from 14.9% for the week ending March 14, 2020, to 1.8% for the week ending April 25, 2020.^6^ The stay-at-home order issued in the state of Illinois on March 9, 2020, also may have played a role in the reduction of seasonal respiratory viral transmission.^7^ During seasons of low rates of respiratory viral transmission, our data suggest that testing for other viruses among COVID-19 patients may not be warranted.

A recent review suggested that antibiotic usage in COVID-19 patients reached 72%.^8^ Antibiotic prescription rates in our pediatric cohort were much lower, suggesting that bacterial coinfection is less likely in pediatric COVID-19 patients. Additionally, antibiotic indications were rarely for lower respiratory tract infection. The most common indication was streptococcal pharyngitis, which may have been incidental.

The limitations of this study include the lack of stratification between inpatients and outpatients, as well as stratification between different age groups because both may impact coinfection rates.

Our findings suggest that viral coinfections in pediatric patients with SARS-CoV-2 likely correlate with general respiratory infection rates. However, more longitudinal studies that span the entire viral respiratory season are needed to clarify the rate of secondary infections in this population.
